# Hands On Biofilm! A multidisciplinary public engagement event using kombucha tea pellicle as an accessible example of biofilm

**DOI:** 10.1016/j.bioflm.2023.100169

**Published:** 2023-12-06

**Authors:** Joanna Verran, Jane Wood, James Redfern, Haleh Moravej, Natascha Radclyffe-Thomas

**Affiliations:** a Department of Life Science, Manchester Metropolitan University, Manchester, M1 5GD, UK; b Textile and Fashion Technology, University of Manchester, Manchester, UK; c Department of Natural Sciences, Manchester Metropolitan University, Manchester, M1 5GD, UK; d Department of Health Professions, Manchester Metropolitan University, Manchester, UK; e British School of Fashion, Glasgow Caledonian University London, UK

## Abstract

Public engagement with science has become increasingly important for the scientific community. There are many documented public engagement events that focus on aspects of microbiology, but relatively few utilise biofilms as a topic, despite their importance. Kombucha tea pellicles are easy to grow biofilms, facilitating their use within the public domain as examples of these complex communities.

The aim of this work was to deliver a public engagement event that introduced visitors to general concepts about biofilm, and applications around sustainability, using kombucha. The event encouraged visitors to: build a biofilm using model clay; inoculate kombucha tea cultures using different incubation conditions, as part of a citizen science experiment to assess impact on pellicle biofilm yield; create garments and drapes on mini-mannequins using dried kombucha pellicle fabric, and demonstrate the range and importance of fermented foods (including kombucha tea), and ‘good bacteria’. Quantitative and qualitative indicators of engagement were built into the activities.

More than 1200 visitors, mainly in family groups, visited the event over a 4-h period. Knowledge of biofilms was low at the beginning of the event. Participation in all activities was high. Indicators of quantitative engagement were impressive, but it was difficult to obtain qualitative evidence other than observations from the delivery team (nineteen members) because of the intensity of the event and volume of visitors.

The event was clearly successful in terms of fulfilment of aims, audience engagement and enthusiasm: the embedded evaluations helped to evidence the impact and reach of the event, enabling confidence in dissemination of good practice in the enhancement of public understanding of the importance of biofilm in general, and kombucha in particular.

## 1 Introduction

Public engagement with science has been increasingly important for conveying the importance of microbiology to a range of audiences, acting as a route for dissemination of research findings [1], enabling contribution from the public to enhance/support research in ‘citizen science’ activities [2], raising awareness of important topics of current concern [[3], [4], [5]], and acquainting audiences with more general aspects of microbiology that are important/interesting [6] and indeed wondrous [7].

The biofilm research community is well aware of the importance of biofilm in all walks of life, but acknowledges that more needs to be done to educate and engage non-scientists, for example around ‘more educational material, position papers and text books’ [8]. The National Biofilm Innovation Centre (NBIC) has begun to collate examples of public engagement activities focusing on biofilms (https://www.biofilms.ac.uk/what-are-biofilms/), and there is a need to ensure that such activities are robust, reproducible and safe, with clearly defined aims, identifiable outcomes and evidence of impact. Without a central store of resources, it is always difficult for researchers to find examples, or inspiration, for their own event planning and delivery – as has been noted previously with regard to AMR [9].

‘Hands-on’ activities are the gold standard for education and for ‘active engagement’ [10]. The mantra of engaging with a student's head (cognitive), hands (practical) and heart (values) is easily translated into events designed to engage with a public audience, enabling consideration of the most effective activities to be implemented [11]. However, few events focusing on microbiology -whether as biofilm or otherwise - can facilitate significant ‘hands-on’ activities, due to health and safety concerns, particularly with potentially high numbers of visitors. Furthermore, there is little opportunity to set up experiments and provide feedback/show results post-incubation, although of course visitors can examine preformed biofilm/images or models, use microscopy [12], or experience virtual reality ‘walk-through’ events [13]. If a biofilm growth experiment is set up (inoculation), then there is a time gap (incubation) before results/observations can be viewed or disseminated. Typically for microbiology hands-on activities that involve inoculation of culture media and monitoring of subsequent growth, results are obtained within 24–48 h – much less that the time required to cultivate biofilm. To overcome difficulties with getting such results to public audiences, we have previously successfully used FlickR to display images of results [12]. Offering face-to-face repeat visits for participants to view results a week (Saturday to Saturday for visitor convenience) after inoculation [4] was not successful.

**Fig. 1:**
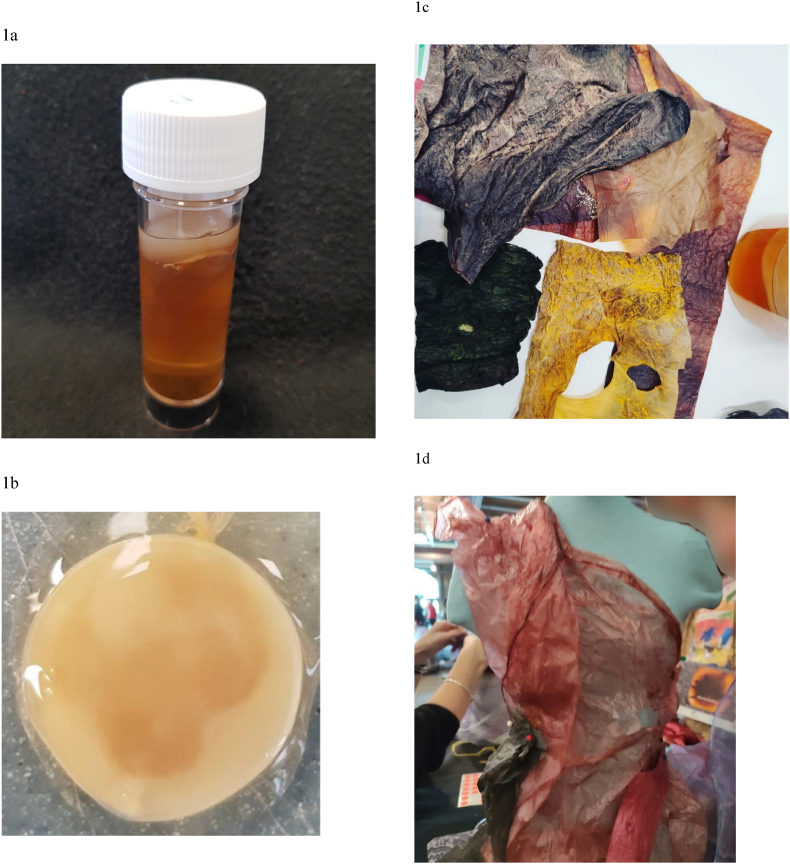
Mature kombucha culture showing the biofilm pellicle on the surface (a), and when decanted from the surface (b). Sheets of kombucha fabric dyed with food dyes (c). Mini-mannequin draped in kombucha fabric (d).

Kombucha pellicle provides an accessible and relatively ‘safe’ biofilm that could be used in a public engagement scenario to generate interest in biofilm more generally whilst also enabling some ‘hands-on’ activities. Kombucha is a fermented, lightly effervescent sweetened black tea drink commonly consumed for its purported health benefits – but it may also be used as a source of biofilm. Kombucha tea can be purchased commercially, but it can also be cultured at home. Home-made Kombucha tea comprises a liquid (the drink) but also a pellicle (biofilm) that grows on the surface of the culture medium (Fig. 1a and b). Both the liquid and the pellicle can be used in subculture to produce more kombucha tea. The global kombucha market is growing exponentially [14], and there is increasing demand for education in the area [15]. It is generally regarded as safe (GRAS) [16], is readily accessible and self-propagating, thus is cost-effective to use, and potentially easily and safely handled. The kombucha pellicle also provides an excellent medium for exploration of biofilm, fermentation and textile technology. Indeed, the pellicle itself has been receiving attention with regard to its textile-like properties when dried, even for its potential use in fashion [17,18], and as a focus for sustainability in the circular economy.

The obvious cross-disciplinary interests that could arise from considering kombucha as a vehicle for public engagement activities (microbiology, nutrition, fermentation, fashion, sustainability) led to the design and delivery of an event that would engage audiences with biofilm, and that included some hands-on experimental activities which incorporated inputs from across disciplines. Repeated exposure to interdisciplinary thought has been shown to enhance learners’ critical thinking and understanding of the relationships between perspectives derived from different disciplines [19], thereby preparing students for dealing with complex societal issues [20]. Using sustainability as an overarching theme for this event provided a focus for the complementarity of the disciplines underpinning the activities provided.

The aim of this paper is to describe and critically evaluate the success of a cross-disciplinary public engagement event designed to raise awareness of biofilms in the context of sustainability and nutrition, using kombucha as an example of biofilm. It is hoped that our experiences might help others planning similar events.

## 2 Methods

The public engagement event was planned for delivery at the 2022 Manchester Science Festival (UK). The annual festival is one of the most popular in the UK, attracting over 100,000 visitors across a week in October. In order to assist those planning to deliver similar events, details about event planning and delivery are included below. It is essential to have identified a location, a target audience and an advertising schedule as part of planning, to ensure that activities, equipment, personnel and consumables are appropriate.

### 2.1 Event planning

#### 2.1.1 Location

Permission to host this event during the Manchester Science Festival (www.scienceandindustrymuseum.org.uk/manchester-science-festival/) was sought and obtained ten months in advance, which enabled a successful funding bid from the National Biofilm Innovation Centre. The one-day event (11am – 3pm), ‘Hands On Biofilm!’, was to be delivered as part of a ‘Get Curious’ stream, which took place daily throughout the ten days of the festival, in the Science and Industry Museum.

#### 2.1.2 Ethics statement

The project was assessed and approved by the Manchester Metropolitan University Ethics Committee (application number 41586) which included the retention of personal data for purposes of contacting visitors after the event. Risk assessment was carried out with input from the host (museum) to assess not only the risk of the activities, but also the practicalities of delivering in an active Museum site. Liability insurance was provided by Manchester Metropolitan University.

#### 2.1.3 Logistics and activities

The delivery team (authors) held several meetings (approximately monthly, more intensively as the event approached) both face to face and online. Ideas and their feasibility (cost, practicality) and fit to the overall aims were discussed. Some were discarded, others used or extended.

In addition to the five academics who hosted the event, several students were recruited to support the activities, comprising seven final year biology project students, five members of MetMUnch, a social enterprise originally run from Manchester Metropolitan University (https://metmunch.com), one MetMUnch intern and one microbiology postgraduate. These were recruited closer to the event and were briefed in the week prior. In addition, the Museum tallied visitor numbers (along with other in-house evaluation).

Previous experience of such events [4,21,22] indicated that a busy day should be anticipated, thus materials required for the event were prepared/sourced with the assumption of 200+ visitors (probably in family groups). Activities needed to be spread out across a designated space near the entrance to the museum, so it was decided to host five stations arranged in an inverted, wide U-shape, each with specific aims, and each with a measure of quantitative engagement embedded. It was hoped that visitors would access the five stations in a specific sequence (left to right), but it was also recognised that this might not happen – so each station delivered independent activities and messages. It was particularly important to ensure that sufficient consumables were available, especially for hands-on activities. Qualitative evaluation would rely on observational conversations, engagement following email follow-up, and critical reflection from the staff and students delivering the event. A hashtag #handsonbiofilm was used to promote and document the event on social media.

### 2.2 Event delivery

Each activity had its own designated space (station). The intended sequence of five stations were set up as listed below, arranged in a ‘7’ shape rather than the ‘U’, due to space constraints in the museum zone: activities began at the foot of the ‘7’. Biofilm-related presenters wore lab coats, MetMUnch staff wore customised aprons.

a) Welcome

**Fig. 2:**
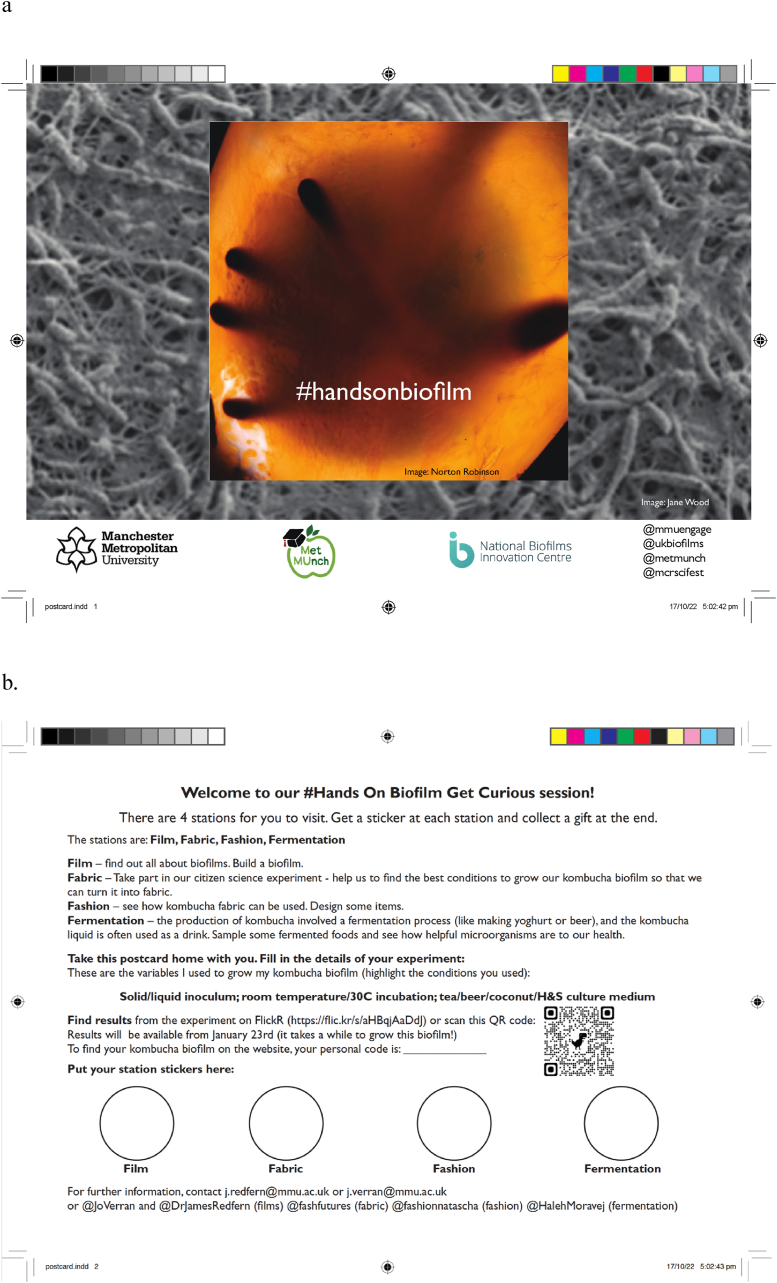
Postcard given to visitors at the Hands On Biofilm event – front (a) and back (b) views. Images courtesy of Jane Wood and Norton Robinson.

One of the authors (JV) took responsibility for the ‘welcome soapbox’. The aim was to encourage engagement with the event, to outline to visitors the various activities taking place, and to provide a postcard with more details (Fig. 2). Stickers were issued at each of the four activities, and if all four stickers were collected on the postcard, then a prize was given. The postcards were to be kept by the visitors so that they could contact the delivery team in future, and use the QR code/FlickR weblink to access a dedicated album where results from the citizen science part of the event would be uploaded. Three hundred postcards were printed. Quantitative evaluation data were obtained from number of postcards distributed, number of stickers collected, and number of visits to the FlickR website.

b) Film

The aim was to inform visitors about biofilm, and to subsequently encourage them to assist in a hands-on experiment using kombucha biofilm.

One of the authors (JR) took overall responsibility for this first visit station, which was delivered primarily by final year undergraduate biology students supported by two of the authors (JV, JR). This station focused on biofilm, and utilised some of the planning for a previous biofilm event planned which was cancelled due to the pandemic.

A rolling gallery of images of biofilms provided a backdrop to the station. Key conversation/information points had been provided to helpers for use in conversation with visitors (what is a biofilm, where do you find biofilm, how do they grow, what do they look like, what does kombucha biofilm look like) in the form of a ‘biofilm explainer’ sheet sourced from NBIC (www.biofilms.ac.uk). Bunchems (www.spinmasters.co.uk), coloured plastic self-adhesive Velcro-like balls (2 cm diameter) were used as visual aids to demonstrate bacterial interactions. Visitors were invited to contribute to our day-long ‘build a biofilm’ activity using Model Magic (www.crayola.com) to make cells that were placed in a home-made Perspex box (415 mm height x 300 width x 35 diameter). To give some indication of prior knowledge, visitors were also asked if they knew what biofilms were, placing a white or clear marble into a jar if they did and other colours if they did not.

Specific risks for this station: Bunchems were not to be used by visitors (risk of tangling in hair); trays and handwipes to be used with Model Magic (to prevent spillage/transfer onto museum floor); supervised use of marbles (choke risk). Quantitative data were obtained from numbers of marbles used to indicate familiarity with biofilm, and number of model magic ‘cells’ made.

c) Fabric

The aim of this part of the day was to get visitors to select inoculation and incubation conditions from choices provided – a further overall aim for the authors was to assess the variability of biofilm yield using different inoculation and incubation conditions, with multiple operators carrying out the process as part of a research project.

One of the authors (JW) took responsibility for setting up the ‘fabric’ station, which was delivered by postgraduate and undergraduate project students and supported by another author (JR). Research in our laboratories had shown that the yield of kombucha pellicle biofilm could vary, and that different incubation conditions were used by researchers in the field [18,23]. After inoculation and five weeks incubation, the biofilm yield would be assessed (thickness, wet and dry weight). [This citizen science activity has been reported in more detail elsewhere [24].]

The postcard provided some general information about the intended activity, but in addition, an A1 poster on a free-standing easel was used to talk visitors through their choices (liquid or solid [pellicle] inoculum; 30 °C or room temperature incubation; beer, tea, coconut milk or H&S [25].

The work benches at the station displayed examples of pre-grown kombucha cultures/pellicles in 5L Kilner jars. 300 lidded plastic Universal bottles (25 mL) were prepared, each containing 20 mL of one of the culture media. After inoculation (by visitors - supervised or by scientists observed by visitors), each inoculation vessel was marked with a reference number corresponding to that on the postcard, and variables were also noted on the postcard (and stickers collected).

At the end of the event, all inoculated vessels were taken to the laboratory for incubation for eight weeks at the selected temperature. After this time, biofilm thickness was measured, the biofilm decanted from the vessel, photographed (wet and dry), weighed, and analysis made of the effect of variables on yield [17] (.). Results were presented on FlickR at a date specified on the postcards. Email addresses were sought from the visitors, if they were happy to provide them, to facilitate contact and a reminder of the FlickR opening date. Risk assessment focused on spillage/slip prevention. Quantitative data were obtained from number of vessels inoculated, number of emails provided (and number of visits to the FlickR website).

d) Fashion

The aim of the activity was to raise awareness of kombucha as a sustainable material, and the importance of sustainable fashion in the circular economy, through conversation and hands-on activities Two of the authors (JW, NR) took responsibility for and delivered the activities at this station. The focus was on creativity and exploring innovative sustainable materials for fashion. Participants were presented with a variety of materials including kombucha biofilm which had been dried and stained with a range of food dyes to produce fabric (Fig. 1c). To create the komucha biofilm fabrics for the activity, a Kombucha starter culture was purchased from a commercial supplier (www.Happykombucha.com). The starter culture (SCOBY = symbiotic culture of bacteria and yeast) weighed 200g, and was supplied in a sealed package containing 100 ml of liquid (green tea). Tea liquid medium was prepared by steeping 4 tea bags in 4l of boiling water. After 15 min the tea bags were removed, 400g sucrose added and the mixture stirred until all the sucrose had dissolved. The mixture was left to cool to room temperature. A 5l kilner jar (www.kilnerjar.com) was submerged for 15 min in sterilising fluid (www.milton-tm.com), prepared according to manufacturers instructions, and removed directly before use. Once at room temperature, the tea liquid medium was decanted into the kilner jar, along with the Kombucha SCOBY and the 100 ml of green tea from the delivery package. The kilner jar was then stored at room temperature for 30 days. This process was repeated 3 times (3 kilner jars prepared in total).

After 30 days, liquid was decanted from the kilner jars as a liquid inoculum for the event. 250 ml aliquots were decanted into sterile containers, and lids screwed shut in preparation for transportation to the event. At this time, a fresh pellicle had developed on the surface of the tea liquid in each of the kilner jars. This pellicle was removed (leaving the original ‘mother’ pellicle in situ) and sliced into 2g pieces as a solid inoculum for the event. The 2g pellicle pieces were stored in a lidded sterile container with 100 ml of the liquid inoculum (decanted from the kilner jar).

The fabrics were of differing texture and thickness, due to variations in growing and drying technique (flat dry on bench vs ‘stretching’ whilst drying, and the addition of coconut oil to some of the fabrics to enhance flexibility). They were dyed using natural food colouring.

Inspired by discovering the tactile qualities of these samples, visitors drew their own designs on paper, which were then converted to clothing by pinning/draping kombucha fabrics onto mini-mannequins (quarter scale size, made at The University of Manchester from foam). No specific risks were identified for this activity. No quantitative data were obtained for this activity.

e) Fermentation

The aim/objective of this station was to inform visitors of the importance of microorganisms in fermented foods and drinks, and in the gut microbiome.

One of the authors (HM) took responsibility for and led the delivery at this station, assisted by a team of undergraduate ‘MetMUnchers’ and an intern. MetMUnch (www.metmunch.com) is very familiar with this type of event, catering for large numbers of visitors at events with various food-related focus. Examples of fermented foods (Kimchi, kefir, kombucha, sauerkraut) were displayed, and samples were available for tasting. MetMUnch members posted images during the event on social media platforms when time allowed.

Leaflets had been produced describing the microbiological activities taking place: 200 copies were made. For visitors who had collected all four stickers, the prize was a bottle of kombucha drink made specifically for the event (www.tigertea.co.uk). Two hundred bottles of kombucha drinks were sourced, with 50 of each flavour (chai, jasmine, earl grey, mojito) being available.

Risk assessment for this station focused on food consumption and disposal of waste.

Quantitative data were obtained from (approximate) number of samples tasted, number of leaflets distributed and number of prizes handed out.

## 3 Results and discussion

### 3.1 Overall observations

Findings from the evaluations carried out at the event are summarised in Table 1. Reflections of the delivery team have been incorporated throughout the results. More than 1200 visitors attended the event (information from the museum), primarily in approximately 300 family groups (observation from stations) – all 300 postcards were distributed throughout the day; all 200 fermentation leaflets were used (with demand for more and promises of digital copies being sent to around twenty additional families). There were 170 kombucha drink bottles given to families who had completed the activities (collecting stickers). The original intention was to have different coloured stickers at each station, to indicate whether any were more, or less, popular. However, the stations were so busy that this organisation could not be maintained: stickers ran out, and the postcards were initialled by those at the stations. The use of ‘hands-on’ activities proved effective in ensuring visitor engagement and enjoyment. This was not merely indicated by the extensive use of materials, but also by the conversations and discussions taking place alongside the varying tasks set [10]. For example, families discussed among themselves and with the demonstrators which experimental conditions to select at the ’fabric’ station, and were keen to carry out the inoculations themselves. The tasting at the ‘fermentation’ station encouraged conversation and comments on flavour and texture, and the time spent on designing outfits and selecting fabrics at the ‘fashion’ station facilitated significant engagement (vide supra). These observations reinforce ‘minds-on’ as well as ‘hands-on’ activities, which will inevitably enhance memory and learning outcomes [11].

**Table 1:** Summary of the four activity stations hosted for the ‘HandsOnBiofilm!’ public engagement event, including aims, methods for quantitative evaluation and general observations.

Activity Station	Aim	Evaluation method	Data/indicator of engagement	Observations
Film	To inform about biofilm.	Question: do you know what biofilm is? Help us build a biofilm.	9 of 174 respondents knew what biofilm was. 530 separate items contributed to built biofilm.	Numerical indicators of engagement supported observed enthusiastic conversations.
Fabric	To select incubation conditions for growing kombucha biofilm. To identify interest in the project over time. To assess the variability of biofilm yield.	Number of vessels inoculated. Visits to website for results posted 12 weeks after the event. Thickness and weight of biofilm measured.	221 vessels inoculated. 4647 views on FlickR website after email reminder. Not directly related to event evaluation, but an embedded citizen science project.	Numerical indicators of engagement supported observed enthusiastic conversations. Importance of obtaining contact details to enhance subsequent engagement. Citizen science project data supported laboratory findings [17].
Fashion	To raise awareness of kombucha as a sustainable material, and the importance of sustainable fashion in the circular economy.	Noting points raised in conversation.	General notes regarding interest and enthusiasm; specific comments not recorded.	Station too busy to formally note observations. Subjective assessment of interactions revealed enthusiastic engagement.
Fermentation	To inform visitors of the importance of microorganisms in fermented foods and drinks, and in the gut microbiome.	Leaflets handed out. Food and drink samples. Noting points raised in conversation.	All 200 leaflets handed out. All foods sampled. General observations regarding enjoyment of taste; specific comments not recorded.	Station too busy to formally note observations. Subjective assessment of interactions revealed enthusiastic engagement.
Event overall	To encourage engagement with the event	Event information provided. Stickers collected from the four stations.	All 300 postcards handed out. 170 groups completed the activities.	1200 visitors in total, mainly in family groups. Quantitative evaluation data indicate successful engagement. Qualitative evaluation was subjective, but indicative of successful engagement.

The event was busy throughout the day without any quieter periods. Student helpers were enthusiastic, informative and knowledgeable, but it was difficult for any of the delivery team to take time away from the event for reflection or observation - even for refreshment. If additional observers had been recruited [3], it is likely they would also have been involved in activities rather than as passive watchers.

Despite the detailed planning and preparation for the event we also needed to be prepared for the unexpected (for example table size was unknown in advance, which affected numbers needed; the station layout needed to be altered on site; sticker provision was inadequate; crowd bottlenecks needed management).It is important to have the support of the establishment hosting the event, and an enthusiastic and robust delivery team. The amount of staff time and effort put into the event was significant and could not have been factored into the funding sought.

### 3.2 Social media

It was barely possible to update social media (Twitter, Instagram or Facebook) about the activities during the event because of the intensity and popularity of visitor interaction. Overall, approximately twenty social media posts received around 300 impressions each. A subsequent thread was posted during World Antimicrobial Awareness Week/National Biofilm Week four weeks after the event. Daily posts throughout the week received around 400 impressions each, although the first post garnered almost 3,000. In hindsight, it might have been preferable to prepare a sequence of timed tweets in advance of the day.

In short, social media proved not to be a useful vehicle for dissemination of this event, particularly in real time, hence the best way of accessing more distant audiences interested in biofilms or public engagement may be specifically designed content for social media as well as remaining with peer-reviewed papers, specific website collections and conference presentations across a range of disciplines.

### 3.3 Film station

The ‘film’ station was the first that most visitors encountered. For the initial question ‘do you know what a biofilm is?’, 174 marbles had been put into the appropriate jar, of which nine were white/clear, indicating that very few visitors knew what biofilm was. However, the jar was placed at the end of the station after visitors had encountered some of the activities: perhaps the responses were related to this immediate prior learning. It would have been better if the question were posed at the initial soapbox introduction, if some follow-up activity had been performed at the end, and if some reflective questions had been included in the subsequent email. Nevertheless, a ‘new’ audience had been reached, for whom biofilm was a novel phenomenon. Often these kinds of events mostly reach people with high prior knowledge, who are already motivated to learn more [26] so the event was of value for enhancing knowledge of an important aspect of microbiology.

**Fig. 3:**
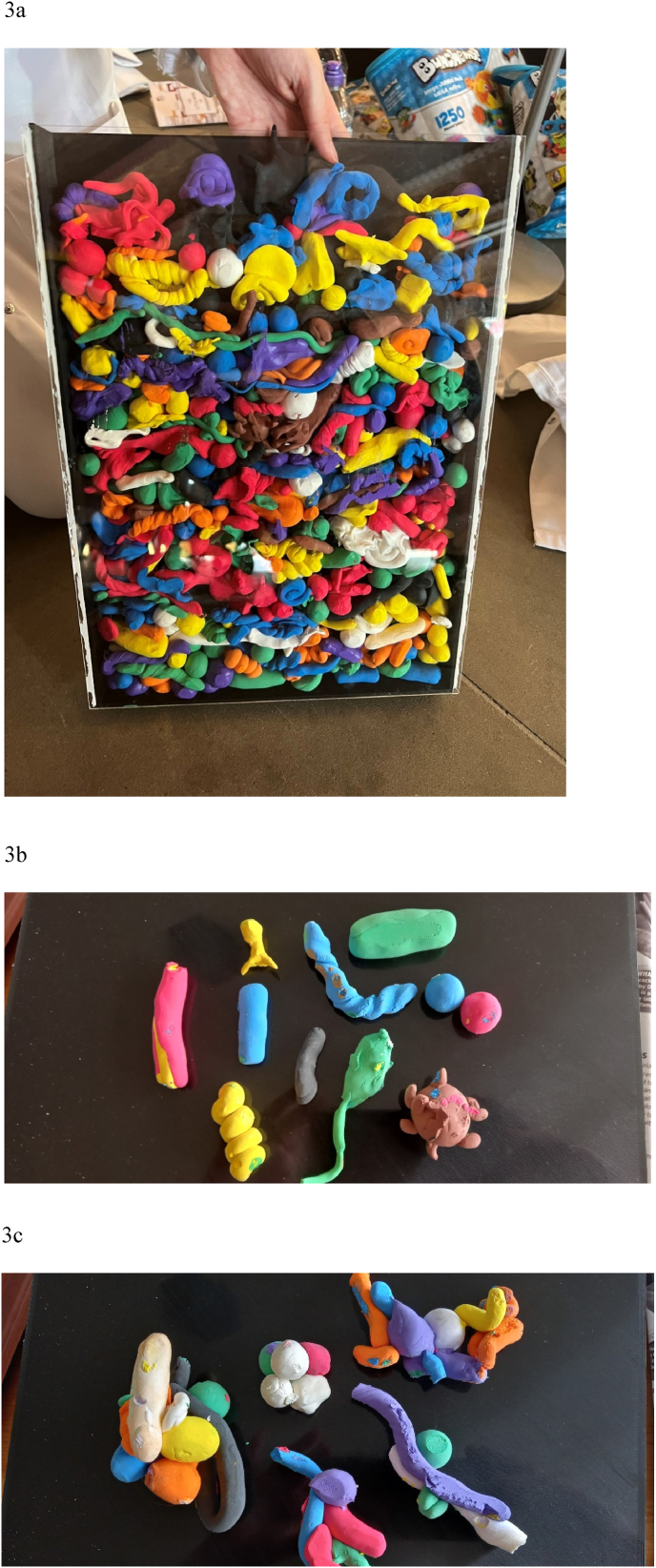
Biofilm models made using Model Magic clay: a) Perspex box containing Model Magic cells as deposited by visitor, b) the diversity of morphological types of ‘microbes’ made by visitors, c) masses of different cell shapes.

The Bunchems were useful as visual aids in demonstrating biofilm formation, but perhaps more reference could have been made to the images on slides, if time and crowds had allowed. The completed Model Magic biofilm comprised 530 separate items/microbial cells (Fig. 3a) not exactly presenting the columnar aspect of a typical biofilm, but reflecting morphological diversity and stimulating engagement. The clay had dried and was very light, but not especially hard, perhaps due to being enclosed in a Perspex box (which nevertheless had to be deconstructed to extract the biofilm). There was a clear diversity of ‘microbial’ shapes (Fig. 3b–c), as well as some models with less of a microbiological flavour (not presented).

### 3.4 Fabric station

The citizen science experiment of growing kombucha pellicles under different incubation conditions generated much interest. There were queues of families waiting to choose their incubation conditions. Details are reported elsewhere [17], but 221 bottles were inoculated, and the FlickR website experienced a total of 4,647 photo views, with 109 ‘photostream’ (individual) visits after email reminders were sent to those 114 visitors who had provided contact details (25 views prior to the reminder). A second reminder was posted more widely on Twitter six months after the event to highlight the results posted on FlickR: within a week, there were an additional 800 views/twenty visitors to the site. It was very rewarding to have evidence of a sustained interest and continued engagement in the event: many evaluations stop when the event closes, which makes evidence of continued interest unusual and valuable. The ‘best’ incubation conditions identified by this citizen science project agreed with those from the research laboratory (tea medium, liquid inoculum, 30 °C or room temperature), supporting the occasionally-disputed fact that citizen science can be on a par with "professional science" [27].

### 3.5 Fashion station

The fashion station was really popular, with queues forming for the budding designers to create their fashion – this activity was more time-consuming than others available to visitors, which also contributed to the bottleneck (at the hinge of the ‘7’). The expectation that all designs would be converted to clothing on the mini-mannequins could not be met (and had not been promised), but some wonderful costumes were created (Fig. 1d). This station could have utilised more tables and had more staff involved. Drawn and coloured designs could have been photographed and counted, but there was no time for quantitative evaluation. Although fashion is frequently considered a female interest, the station host observed that the visitors did not reflect any such stereotypes. The ability to touch materials and see them transformed into 3D ‘garments’ was evidently impactful. Visitors were surprised and intrigued about the potential for such innovative materials. The range of outcomes was varied – from sportswear to couture! Most visitors proudly shared their creations with their families and took photos of their sketches and the finished pieces. It is unfortunate that there were no quantitative indicators of engagement at this station (designs could have been collected or photographed, and/or counted), and that qualitative indicators relied on observations of the experts hosting the activities. However, the popularity of the station was evident from the bustle and crowding around the tables, and the conversations demonstrated the value of using different disciplines to consider the overarching sustainability theme [19].

### 3.6 Fermentation station

The fermentation station was also busy. Some of the aims of the station were more adult-focused, relating to mental health, irritable bowel syndrome issues and food sustainability. However, families and children enjoyed tasting the foods, and appreciated the additional and accessible information provided in the booklet. This combination of fermented food, fundamental microbiology and sustainability have been explored previously in public engagement events [22], illustrating the value of cross-disciplinary collaboration and the ‘head, hands, heart’ – and gut? - concept of active learning [11].

Kimchi was the most popular of the fermented foods (supplies ran out first), Some families did not want to take away the kombucha tea samples that were the gift for completing the exercise, although others were pleasantly surprised at the taste. In hindsight, it would have been better to give children sachets of Model Magic as their gift. By the end of the day, when fewer families had multiple (individual) copies of the postcard, the remaining kombucha bottles were given out freely.

### 3.7 Quantitative and qualitative evaluation indicators

Quantitative evaluation indicated considerable engagement with the science by the audiences. Qualitative indicators rely on our observations: the buzz of activity across the event; the diversity of the morphological forms in the Model Magic biofilm; the thoughtful selection of incubation conditions; the time taken to create fashion designs; the ongoing conversations between family members, and between families and the delivery team. More detailed reporting of these conversations was not possible. A possible addition could have been a separate ‘vox-pop’ kiosk for recording visitors views. It would also have been interesting to know how long individual families stayed: certainly the high number of completed postcards/collected stickers indicated that most families (perhaps an estimate of 170 of 300 in total) visited all stations. This observation reinforces the value of multi-disciplinary inputs to public engagement events.

### 3.8 Concluding comments

This was a multi-disciplinary event designed to engage family audiences with kombucha biofilm, comprising activities that crossed subject boundaries, demonstrating the value of collaboration and cross-disciplinarity in science and the arts, with an overarching sustainability perspective. There should have been something for every visitor somewhere across the stations, whether it was good bacteria, sustainable fashion, fermentation or biofilm. Quantitative evaluation indicated successful engagement. Subjective observations of the audience enthusiasm and engagement show that positive messages about science, scientists and universities – and biofilms, textiles, fashion and fermentation – were transmitted for that day at least, and likely beyond, when the citizens got their ‘HandsOnBiofilm’.

## CRediT authorship contribution statement

**Joanna Verran:** Conceptualization, Funding acquisition, Methodology, Project administration, Supervision, Writing – original draft. **Jane Wood:** Conceptualization, Methodology, Project administration, Resources, Supervision, Writing – review & editing. **James Redfern:** Conceptualization, Funding acquisition, Methodology, Project administration, Resources, Supervision, Writing – review & editing. **Haleh Moravej:** Conceptualization, Funding acquisition, Methodology, Project administration, Resources, Supervision. **Natascha Radclyffe-Thomas:** Conceptualization, Funding acquisition, Methodology, Project administration, Resources, Supervision.

## Declaration of competing interest

The authors declare the following financial interests/personal relationships which may be considered as potential competing interests: James Redfern, Joanna Verran reports financial support was provided by National Biofilms Innovation Centre.

## Data Availability

No data was used for the research described in the article.
